# Age group differences in association between IADL decline and depressive symptoms in community-dwelling elderly

**DOI:** 10.1186/s12877-019-1333-6

**Published:** 2019-11-13

**Authors:** Eri Kiyoshige, Mai Kabayama, Yasuyuki Gondo, Yukie Masui, Hiroki Inagaki, Madoka Ogawa, Takeshi Nakagawa, Saori Yasumoto, Hiroshi Akasaka, Ken Sugimoto, Kazunori Ikebe, Yasumichi Arai, Tatsuro Ishizaki, Hiromi Rakugi, Kei Kamide

**Affiliations:** 1 Department of Health Promotion Science, Osaka University, Graduate School of Medicine, Osaka, Japan; 2 Japan society for the protion of science, Tokyo, Japan; 3 Department of Clinical Thanatology and Geriatric Behavioral Science, Osaka University Graduate School of Human Sciences, Osaka, Japan; 4 Tokyo Metropolitan Institute of Gerontology, Research Team for Human Care, Tokyo, Japan; 5 Section of NILS-LSA, National Center for Geriatrics and Gerontology, Aicehi, Japan; 6 Department of Geriatric and General Medicine, Osaka University Graduate School of Medicine, Osaka, Japan; 7 Department of Prosthodontics, Gerontology and Oral Rehabilitation, Osaka University Graduate School of Dentistry, Osaka, Japan; 8 Center for Supercentenarian Medical Research, Keio University School of Medicine, Tokyo, Japan

**Keywords:** Instrumental activities of daily living, Depressive symptoms, Age group, Community-dwelling people, Older adults, Multiple group analysis

## Abstract

**Background:**

Instrumental Activities of Daily Living (IADL) is an indicator of whether a community-dwelling elderly can live independently. IADL decline was reported to be associated with aging and depression. The present study aimed to investigate whether the association between IADL decline and depressive symptoms differs with aging, using two age groups of community-dwelling Japanese elderly in their 70s and 80s.

**Methods:**

We conducted longitudinal analysis among participants in their 70s and 80s at the baseline from Septuagenarians, Octogenarians, Nonagenarians Investigation with Centenarians (SONIC) study. IADL was assessed by The Tokyo Metropolitan Institute of Gerontology (TMIG) index of competence. As a main predictor, depressive symptoms were measured by the five-item version of the Geriatrics Depression Scale (GDS-5). As possible confounders, we considered cognitive function, body mass index, solitary living, education, economic status, medical history of stroke and heart disease, hypertension, dyslipidemia, diabetes, and sex. We obtained odds ratios (ORs) of IADL decline for having depressive symptoms in each age group (70s/80s) and tested interactions between depressive symptoms and age groups in relation to IADL decline in 3 years by logistic regression. Additionally, to confirm age group differences, we conducted multiple group analysis.

**Results:**

There were 559 participants in their 70s and 519 in their 80s. Compared to participants without depressive symptoms, those with depressive symptoms had higher OR of IADL decline in 70s (OR [95% CI] = 2.33 [1.13, 4.78]), but not in 80s (OR [95% CI] = 0.85 [0.46, 1.53]). There were significant interactions between depressive symptoms and age groups in relation to IADL decline (*p*-value = 0.03). Multiple group analyses showed differences between the age groups by Akaike information criterion (AIC), and ORs (95%CI) decline for depressive symptoms was 2.33 (1.14, 4.77) in 70s and 0.85 (0.47, 1.54) in 80s.

**Conclusion:**

The association of depressive symptoms and IADL decline during the 3 years was significantly different between the 70s and 80s age groups, and significant association was found only in people in their 70s. Detecting depressive symptoms may be a key for preventing IADL decline in people in their 70s and not for those in their 80s.

## Background

Among the population aged 65 years and older, 35% of people suffer from disability (e.g., activities of daily living (ADL) decline and instrumental ADL (IADL) decline) [1], which can make it difficult for people to live independently. IADL represents abilities for using public transportation, shopping for daily necessities, preparing meals, paying bills, managing a bank account, and so on [2], meaning that IADL is a key factor for the elderly to live independently, socially, and healthily.

Depressive symptom is an important risk factor for IADL decline [3, 4]. A previous epidemiological study has reported that depressive symptoms increased the risk of difficulties in ADL and IADL [3] among community-dwelling Japanese elderly people aged 75 years and older (*n* = 581). Another previous study using a prospective cohort design has shown that depressive symptoms predicted IADL decline in 3–4 years [4] among people aged 65 years and older in the United States of America (*n* = 3052).

However, the association between depressive symptoms and IADL decline may be different across the age groups of those in their 70s and 80s because of the possible following reasons. The prevalence of impaired IADL in people aged 80 years and over was almost twice than that of people aged 70 to 79 years [5]. Additionally, the prevalence of depression in people aged 85 years and over was twice than that in people aged 70 to 74 years [6]. Finally, a previous cross-sectional research of which participants were community-dwelling adults aged 57 to 99 years in Netherlands (*n* = 5279) reported that depression was significantly associated with IADL decline in people aged 65 to 74 years old (*P* = 0.01), but not significantly in people aged 75 years and over (*P* = 0.07) [7]. However, few studies have investigated whether the association of depressive symptoms with IADL decline was different between age groups.

The present study aimed to investigate whether the association between IADL decline and depressive symptoms was different between the age groups, 70s and 80s, in community-dwelling Japanese elderly.

## Methods

### Participants

This paper used a longitudinal design (3-year follow-up period) from the Septuagenarians, Octogenarians, Nonagenarians Investigation with Centenarians (SONIC) study, an ongoing study since 2010. Participants were selected from urban and rural areas in Western and Eastern Japan: Itami City (urban) and Asago City (rural) in Hyogo prefecture (Western Japan); Itabashi Ward (urban) and Nishitama District (rural) in Tokyo prefecture (Eastern Japan). We sent invitation letters of participation in the SONIC study to residents between 2010 and 2013, of which 1000 participants were aged 69–71, 973 aged 79–81, and 272 aged 89–91 years, with follow-ups of 3-year intervals. This study was approved by the Institutional Review Board of Osaka University Graduate School of Medicine, Dentistry and Human Sciences (Osaka, Japan) and the Tokyo Metropolitan Geriatric Hospital and Institute of Gerontology (Tokyo, Japan). All participants provided written informed consent to participate.

The eligibility criteria for the present paper were the participants of the SONIC study 1) who were 69–71 years old in 2010 as the baseline for the 70s age group, and who were aged 79–81 years old in 2011 as the baseline for the 80s age group, 2) whose IADL were not declining and were a full score at the baseline, and 3) who participated in the SONIC study at the baseline and periods of the 3-year follow-ups. Participants with missing information on IADL and depressive symptoms were excluded (122 for 70s, and 51 for 80s). The number of participants of the present paper was 1078, consisting of 539 in the 70s group and 519 in the 80s group.

### The IADL assessment

In the present study, IADL was assessed by an IADL subscale, consisting of five questions from The Tokyo Metropolitan Institute of Gerontology (TMIG) index of competence [2]. Participants answered each question with “yes” or “no” indicating whether or not the individual is able to do a particular activity (i.e. using public transportation, shopping, food preparation, paying bills, handling bank account). The number of items answered “yes” indicated the total score of the IADL subscale, ranging from 0 (worst) to 5 (best). We considered participants whose IADL scores were 0–4 as participants with IADL decline [8]. We assessed the IADL at baseline and at periods of the 3-year follow-ups.

### Depression symptoms

To assess depressive symptoms, we used the five-item version of the Geriatric Depression Scale (GDS-5) [9]. Participants answered each question with “yes” or “no”, providing a total GDS score of 0–5; a higher score indicated a more severe depressive state. Participants with a total GDS-5 score < 2 were defined to have non-depressive symptoms, and those with a total GDS-5 score≧2 were defined to have depressive symptoms [10]. The cut-off point for depressive symptoms has been reported to have a sensitivity of 0.97 and specificity of 0.85 for depressive symptoms assessed by Diagnostic and Statistical Manual of Mental Disorders, Fourth Edition (DSM-IV) criteria [9]. We also used the cut-off point of the GDS-5, and categorized the present participants as having either non-depressive or depressive symptoms.

### Possible confounders

As possible confounders, the following variables were considered: sex, cognitive function, economic condition, education, solitary living, histories of stroke and heart disease, hypertension, dyslipidemia, diabetes, and body mass index (BMI), which were based on previous studies of the associations between depressive symptoms [11, 12] and IADL decline.

In the present study, cognitive function was assessed by the Japanese version of the Montreal Cognitive Assessment (MoCA-J) [13]. The MoCA-J test is a validated tool used to assess global cognition and was developed to assist in the diagnosis of MCI [14]. Participants take approximately 10 min to take the MoCA-J test. The MoCA-J assesses seven cognitive functions: visuospatial ability (3 points), naming task (3 points), attention task (6 points), language (3 points), abstraction task (2 points), delayed recall (5 points), and orientation (6 points). Ranging from a total of 0 to 30 points, a lower score indicated a more sever impairment of cognitive function.

Height was measured using calibrated height meters to the nearest 0.1 cm while the participant stood erect without shoes. This measurement was then converted to meters. Weight was measured using calibrated body weight machine to the nearest 0.1 kg in light clothing without shoes. BMI was calculated as the weight in kilograms divided by the square of the height in meters (kg/m^2^). Hypertension was defined by medication use for hypertension or BP level (i.e., systolic BP > 150 mmHg or diastolic BP > 90 mmHg). To measure the BP levels, physicians and trained nurses used the mercury sphygmomanometer. BP was measured twice on each arm in a sitting position. The average of the first and second measurements on each arm was used for analysis. The present study defined dyslipidemia by medication use of dyslipidemia, low-density lipoprotein cholesterol≧140 mg/dL, high-density lipoprotein cholesterol< 40 mg/dL, or triglyceride≧150 mg/dL. In the present study, diabetes was defined by medication use of diabetes, hemoglobin A1c≧7.0%, or casual plasma glucose concentration≧200 mg/dL. Those definitions were based on the criterion of Japanese guidelines for aged people [15–17]. Histories of stroke and heart disease were collected by a self-reported questionnaire answered by “yes” or “no” in health check-ups.

We categorized education years into two categories (< 16 years / ≥ 16 years) corresponding to graduation from University or not. Economic condition was based on subjective satisfaction of household income by three options (Dissatisfying, Neutral, Satisfying) in the questionnaire, which we further categorized into two for analysis (Dissatisfying / Neutral or Satisfying). We gained information about solitary living from the question, “who do you live with?” This was answered by one of five options (No one, Spouse, Parents(−in-law), Children(−in-law), Others). We grouped these answers into two categories for analysis to indicate solitary living by the option “No one” and non-solitary living by all the other options.

### Statistical analyses

To investigate the association between depressive symptoms and IADL decline in 3 years, we conducted logistic regression models stratified by the age groups (70s / 80s) to obtain odds ratios (ORs) and 95% confidence intervals (CI). For this we used two models. In the first model (Model 1), the association of depressive symptoms with IADL decline was adjusted for sex (male / female) and cognitive function assessed by the MoCA-J. In the second model (Model 2), the association was adjusted for sex, cognitive function, BMI, histories of stroke (yes/no) and heart disease (yes/no), solitary living (yes/no), economic condition (Dissatisfying / Neutral or Satisfying), education (≦16 years / > 16 years), hypertension (yes/no), dyslipidemia (yes/no), and diabetes (yes/no).

To investigate whether the association of the baseline depressive symptoms with IADL decline in 3 years was different between the two age groups of 70s and 80s, we investigated interactions between depressive symptoms and age groups in relation to IADL decline in 3 years by logistic regression analyses and also conducted multiple group analysis. Multiple group analysis is an analysis of a framework of structural equation modeling method. This analysis can estimate simultaneously across groups [18, 19]. We compared an equality constrained model with an unconstrained model to investigate whether the association of depressive symptoms with IADL decline was different between the age groups (i.e., 70’s and 80’s). In an equality constrained model, regression coefficients of depressive symptoms are estimated to be equal between the age groups. On the other hand, in an unconstrained model, regression coefficients of depressive symptoms are freely estimated across the age groups. We compared those two models by Akaike information criterion (AIC), a good-of-fit index with considering the rule of parsimony. When the unconstrained model is better than the equality constrained model, we can interpret that the regression coefficients of depressive symptoms are not equal across the age groups, meaning that the associations between depressive symptoms and IADL decline are significantly different between the age groups. On the other hand, when the equality constrained model is better, we can interpret that the regression coefficients of depressive symptoms are equal across the groups, meaning that the associations are not significantly different between the groups. In the present study, we set estimates of regression coefficients of depressive symptoms on the IADL decline equal between 70s age groups and 80s age groups in the equality constrained model. We obtained odds ratios (ORs) and 95% confidence intervals (CI).

Logistic regression analysis was performed by statistical software R version 3.4.0. In addition, Multiple group analysis was conducted by Mplus version8.3 [20].

## Results

**Fig. 1:**
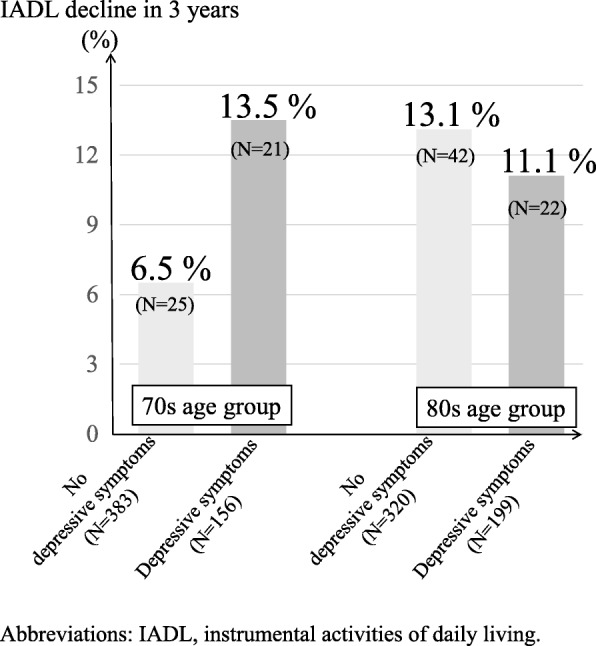
Proportions of IADL decline in 3 years stratified by age groups (70s and 80s) and depressive status. Abbreviations: IADL, instrumental activities of daily living

Of the eligible 1078 participants, 156 (28.9%) and 199 (38.3%) participants suffered from depressive symptoms in the 70s and 80s groups, respectively. The baseline characteristics of the present participants were summarized in Table 1. In the 70s group, of the 156 participants with depressive symptoms, 21 (13.5%) had IADL decline in 3 years, and of the 383 participants without that, 25 (6.5%) had IADL decline. In the 80s group, of the 199 participants with depressive symptoms, 22 (11.1%) had IADL decline in 3 years, and of the 320 participants without that, 42 (13.1%) had IADL decline. (Fig. 1).

**Table 1:** Baseline characteristics of the participants based on the SONIC study stratified by age groups (70s and 80s) and depressive status

	70s age groups		80s age groups	
	without depressive symptoms	with depressive symptoms	without depressive symptoms	with depressive symptoms
n (%)	383 (71.1)	156 (28.9)	320 (61.7)	199 (38.3)
Female	206 (53.8)	75 (48.1)	174 (54.4)	108 (54.3)
IADL decline in 3 years	25 (6.5)	21 (13.5)	42 (13.1)	22 (11.1)
History of stroke	16 (4.2)	9 (5.8)	14 (4.4)	13 (6.8)
History of heart disease	51 (13.5)	19 (12.2)	49 (15.5)	38 (19.7)
Hypertension	243 (63.8)	100 (64.1)	236 (73.8)	149 (74.9)
Dyslipidemia	227 (60.5)	97 (64.2)	191 (59.7)	131 (65.8)
Diabetes	34 (9.7)	19 (13.7)	35 (11.4)	15 (7.9)
Dissatisfaction of household income	63 (16.4)	42 (26.9)	39 (12.2)	44 (22.1)
Education of less than 16 years	317 (82.8)	133 (85.8)	276 (86.5)	163 (81.9)
Solitary living	45 (11.7)	23 (14.7)	69 (21.6)	53 (26.6)
Mean (SD)				
MoCA-J score	24.0 (3.2)	23.7 (2.9)	22.6 (3.4)	22.3 (3.4)
Body Mass Index (kg/m2)	23.0 (2.8)	22.8 (3.1)	22.4 (2.8)	22.9 (4.4)

*Abbreviations*: *IADL* Instrumental activities of daily living, *MoCA-J* Japanese version of the Montreal Cognitive Assessment

We investigated the association of depressive symptoms with IADL decline in 3 years for the 70s age group and the 80s age group (Table 2). IADL decline was significantly associated with depressive symptoms in the 70s age group, but not in 80s. When sex and cognitive function were adjusted, ORs (95%CI) of IADL decline for having depressive symptoms compared with not having depressive symptoms were 2.11 (1.10, 4.03) in 70s and 0.81 (0.46, 1.40) in 80s. We obtained similar results in model 2 as a fully-adjusted model. Additionally, we found significant interactions between depressive symptoms and age groups in relation to IADL decline: those *p*-values were 0.02 in Model1 and 0.03 in Model2 (Table 2).

**Table 2:** Adjusted OR (95% CI) and Mean (95% CI) of IADL decline in 3 years for depressive symptoms obtained by multiple logistic regression models

		Model1^b^		^a^*P*-values for interaction	Model2^c^		^a^*P*-values for interaction
		70s age group	80s age group		70s age group	80s age group	
	ORs (95% CI)						
Depressive symptoms	No	1 (Ref.)	1 (Ref.)	0.02	1 (Ref.)	1 (Ref.)	0.03
	Yes	2.11 (1.1, 4.03)	0.81 (0.46, 1.4)		2.33 (1.13, 4.78)	0.85 (0.46, 1.53)	
Sex	Male	1 (Ref.)	1 (Ref.)		1 (Ref.)	1 (Ref.)	
	Female	0.14 (0.05, 0.31)	0.53 (0.31, 0.91)		0.13 (0.04, 0.32)	0.58 (0.31, 1.08)	
	Mean (95% CI)						
MoCA-J score		0.85 (0.77, 0.94)	0.97 (0.90, 1.05)		0.87 (0.78, 0.97)	0.98 (0.9, 1.07)	
Body Mass Index (kg/m2)		–	–		1.05 (0.92, 1.19)	0.99 (0.9, 1.06)	
	ORs (95% CI)						
History of stroke	No	–	–		1 (Ref.)	1 (Ref.)	
	Yes	–	–		2.17 (0.53, 7.41)	0.62 (0.1, 2.25)	
History of heart disease	No	–	–		1 (Ref.)	1 (Ref.)	
	Yes	–	–		1.29 (0.49, 3.12)	0.91 (0.4, 1.89)	
Hypertension	No	–	–		1 (Ref.)	1 (Ref.)	
	Yes	–	–		0.89 (0.41, 2.01)	0.77 (0.43, 1.45)	
Dyslipidemia	No	–	–		1 (Ref.)	1 (Ref.)	
	Yes	–	–		0.61 (0.30, 1.25)	0.68 (0.38, 1.21)	
Diabetes	No	–	–		1 (Ref.)	1 (Ref.)	
	Yes	–	–		1.3 (0.44, 3.39)	1.26 (0.48, 2.88)	
Household income	Satisfaction to neutral	–	–		1 (Ref.)	1 (Ref.)	
	Dissatisfaction	–	–		1.24 (0.52, 2.78)	1.38 (0.65, 2.76)	
Education	≧ 16 years	–	–		1 (Ref.)	1 (Ref.)	
	< 16 years	–	–		2.44 (0.92, 7.71)	1.28 (0.57, 3.17)	
Solitary living	No	–	–		1 (Ref.)	1 (Ref.)	
	Yes	–	–		0.18 (0.01, 0.95)	1.05 (0.50, 2.11)	

Because of missing information of possible confounders, these logistic regression analyses were analyzed by using 537 participants in 70s and 518 in 80s in Model 1, and 482 in 70s and 483 in 80s in Model 2

^a^Interaction between depressive symptom and age groups (70s/80s). 70s and 80s age groups were combined at the same time to test the interaction

^b^Model 1 adjusted for sex and MoCA-J score

^c^Model 2 adjusted for sex, MoCA-J score, Body Mass Index, histories of stroke and heart disease, economic condition, education, hypertension, hyperlipidemia, and diabetes

*Abbreviations*: *OR* Odds ratio, *CI* Confidence interval

To focus on the age group differences (70s/80s) for the association, we conducted multiple group analysis (Table 3). Compared with the unconstrained models, the equality models were worse based on AICs (2128 for equity vs 2125 for unconstrained in Model 1 and 1961 vs 1964 in Model 2). For unconstrained model in Model 1, ORs of IADL decline for depressive symptoms compared with no having depressive symptoms were 2.11 (1.11, 4.03) in 70s and 0.81 (0.47, 1.41) in 80s. We obtained similar results in model 2 as a fully-adjusted model (ORs = 2.33 95%CI, 1.14, 4.77 in 70s; ORs = 0.85 95%CI, 0.47, 1.54 in 80s).

**Table 3:** ORs (95%CI) and AIC obtained by multiple group analyses in 70s age group (*N* = 539) and 80s age group (*N* = 519)

	Model^1^		Model2^b^	
	Unconstrained model	Equality constrained model	Unconstrained model	Equality constrained model
ORs (95%CI)				
Depressive symptoms				
70s age group	2.11 (1.11, 4.03)	1.20 (0.79, 1.82)^c^	2.33 (1.14, 4.77)	1.27 (0.81, 2.00)^c^
80s age group	0.81 (0.47, 1.41)		0.85 (0.47, 1.54)	
Fit indices				
AIC	2125	2128	1961	1964

Because of missing information of possible confounders, these multiple group analyses were analyzed by using 537 participants in 70s and 518 in 80s in Model 1, and 482 in 70s and 483 in 80s in Model 2

^a^Model1 adjusted for sex and MoCA-J score

^b^Model2 adjusted for sex, MoCA-J score, Body Mass Index, histories of stroke and heart disease, economic condition, education, hypertension, dyslipidemia, and diabetes

^c^Equality constrained model was constrained with equality constrained across age groups

*Abbreviations*: OR Odds ratio, *CI* Confidence interval, *RMSEA* Root mean square error of approximation, *AIC* Akaike information criterion

## Discussion

The present study aimed to investigate whether the association of depressive symptoms with IADL decline was different in elderly people in their 70s and in their 80s. Logistic regression analysis showed that depressive symptoms were significantly associated with IADL decline in 70s (OR = 2.33, 95%CI, 1.13, 4.78), but not in 80s (OR = 0.85, 95%CI, 0.46, 1.53). Additionally, we found significant interaction between depressive symptoms and the age groups in relation to the IADL decline (*p* = 0.03). Furthermore, the multiple group analyses showed that, the unconstrained model was better than the equality constrained model for the association based on AIC, meaning there was different between 70s and 80s on the association of depressive symptoms with the IADL decline.

The present result of the significant association of depressive symptoms with IADL decline in the 70s group by logistic regression and multiple group analysis were similar to the previous study [21]. The present results might be due to the following possible reasons. According to a previous study [21], depressive symptoms such as fatigue, sleep disturbance, and loss of appetite could reduce motivation for treatments and preventions of disabilities. Additionally, people with depressive symptoms were also undermined their resistance to disabilities including IADL decline [21].

The present study showed significant association of depressive symptoms with IADL decline in the 70s group but not in the 80s. Possible reasons for this could be as follows. First, in a previous study [22], depression was found to be common among people aged 85 years and over; its prevalence was 15.3% [6]. The participants’ age in this previous study was similar to that in the present study. Second, the proportion of solitary living was high only in the 80s age group with depressive symptoms in the present study (Additional file 1: Table S1). People living alone in communities may not need significant help from other people and social services, suggesting that their IADL abilities were relatively high. In fact, a previous epidemiological study showed that people with depressive status had significantly lower scores of IADL compared to those without depressive status, which was clearer in people aged 65 to 74 years (*p* = 0.01) than in people aged 75 years and older (*p* = 0.07) [7]. However, this particular study did not aim to detect differences between the age groups on the association, and it did not conduct a statistical test for the possible age-group differences. On the contrary, the present study directly compared the possible difference between age groups, specifically 70s and 80s, on the association of depressive symptoms with IADL decline within 3 years by multiple group analysis which a significant difference. Additionally, the present study used a longitudinal design, though the previous study used a cross-sectional design.

In the present study, we could suggest the association between depressive symptoms and IADL decline may change by the age groups, 70s and 80s. Health professionals should take care of IADL decline when their patients in their 70s have depressive symptoms. On the other hand, health professionals should pay attention to other potential risks (e.g., cognitive decline) rather than IADL decline even when their patients in their 80s have depressive symptoms.

### Strengths and limitations

We had the following strengths. We directly investigated differences between the two age groups on the association of depressive symptoms with IADL decline because previous studied did not investigate the possible differences even though prevalence of depression symptoms and IADL decline were different between the age groups. The present study used a longitudinal design, which allowed us to decide temporal order on the association of depressive symptoms with IADL decline.

Nevertheless, the present study had the following limitation. First, in the present study, IADL and depressive symptoms were assessed by a self-reported questionnaire. IADL was assessed by TMIG index of competence. However, reliability coefficients indicated high reliability of the TMIG Index of Competence (the reliability coefficient Alpha was 0.91; the 1-year test-retest reliability coefficient was 0.86) [2].

Additionally, depressive symptoms were assessed by GDS-5 that was a self-reported questionnaire. However, GDS-5 had a sensitivity of 0.97 and a specificity of 0.85 for depressive symptoms assessed by Diagnostic and Statistical Manual of Mental Disorders, Fourth Edition (DSM-IV) criteria [10]. Second, there could be residual confounding factors such as marital status and occupation. However, the present study considered the following possible confounders: sex, age, cognitive function, body mass index, histories of stroke and heart diseases, economic condition, education, hypertension, dyslipidemia, and diabetes mellitus.

## Conclusion

The present study showed that the association of depressive symptoms with IADL decline within 3 years was different between the age groups of 70s and 80s. Interestingly, the 70s age group had a significant association, while the 80s did not. Prevention of IADL decline should be planned considering the age group; for example, detecting depressive symptoms may be a key for preventing IADL decline in elderly people in their 70s and not for those in their 80s. Health professionals should pay attention to other risks related to aging rather than IADL decline when their patients in their 80s have depressive symptoms.

## Supplementary information

**Additional file 1: Table S1.** Adjusted Odds Ratios (95%CI) of Depressive symptoms for each baseline characteristic stratified by age groups (70s and 80s) and depressive status.

## Data Availability

The datasets used and/or analyzed during the current study available from the corresponding author on reasonable request.

## Abbreviations

AIC: Akaike information criterion
CI: Confidence interval
IADL: Instrumental activities of daily living
MoCA-J: Japanese version of the Montreal Cognitive Assessment
OR: Odds ratio
RMSEA: Root mean square error of approximation
